# Influence of the Urban Built Environment on Physical and Mental Health of the Elderly under the Background of Big Data

**DOI:** 10.1155/2022/4266723

**Published:** 2022-07-21

**Authors:** Shuting Yan, Lei Shi, Li Wang

**Affiliations:** ^1^School of Architecture and Art, Central South University, Changsha 410075, China; ^2^Hunan Open University, Changsha 410075, China; ^3^China Machinery International Engineering Design and Research Institute Co., Ltd, Changsha 410075, China

## Abstract

With the advent of the information technology revolution and the Internet era, information technology is gradually occupying an important position and becoming an important strategic factor in economic development. As an emerging technology that has been developing continuously in recent years, big data is becoming an important industry to improve the innovation and development of the urban economy. Like AI technology, cloud computing, and the Internet, big data has become an important application technology for economic growth and economic efficiency improvement in today's world. It is an effective means of progress and development in a region and an important strategic resource. As a new technology, big data has attracted more and more attention from all walks of life. Many companies have turned their attention to developing big data for economic benefits. “Enjoy your old age” is the yearning of every old man and his family. In recent years, the national level has been committed to “creating an urban built environment for the elderly to achieve healthy aging.” From the perspective of promoting the physical and mental health of the elderly, this paper analyzes the impact of the urban built environment on the physical and mental health of the elderly based on the needs of the elderly and puts forward countermeasures and suggestions based on the current status and existing problems of the urban built environment for the elderly. Based on the combined data analysis method and technology in big data, this paper conducted a field questionnaire survey on a total of 4,000 elderly people in urban and rural areas by means of the questionnaire survey. It is found that the existing problems of the built environment in the old cities include scattered content, one-sided understanding, and rigid design. According to the problems, the solutions of building consensus, paying attention to planning, combining urban characteristics, and the joint efforts of all sectors of society are put forward. And programming tools are used to combine formulas and analyze related data in detail. The analysis results show that the physical and mental health index of the elderly is highly correlated with factors such as changes in the consensus degree of the urban built environment, urban built environment planning, urban built environment policy support, and multiparty efforts in the urban built environment. Changes show a positive change.

## 1. Introduction

### 1.1. Physical and Mental Health of the Elderly

At present, the aging phenomenon in China is becoming more and more serious, and it has become one of the hot social issues to be solved urgently. The physical and mental health of the elderly has received extensive attention from all walks of life. The so-called “health” in today's society is not only the soundness of various organs and functions of the body but also the calm and stable psychological state [1, 2]. According to the World Health Organization, the health of older persons is a resource for families, communities, and economies, and a cornerstone of an individual's continued contribution to society. Promoting the health of the elderly is also one of the important goals of the urban built environment for the elderly in various countries [3, 4]. The negative environment may affect the physical and mental health of the elderly. For example, being in a noisy environment for a long time may cause hearing problems and sleep disorders for the elderly. In addition, being in a more fashionable environment will also make the elderly feel unnatural. This paper aims to study the impact of the urban built environment on the physical and mental health of the elderly and then make recommendations.

### 1.2. Big Data Analysis

Today, Internet technology has been rapidly popularized in all walks of life, and it is transforming into an information society at an alarming rate. In addition to the elements of natural, capital, and human resources, big data has become the fourth factor of production. These data provide a platform for people to better understand the world [5–7]. Today, coal, oil, and other resources no longer have absolute development advantages, and the advent of the era of science and technology will make big data an important strategic resource. Although the development of big data in foreign regions is relatively mature, it is still in its infancy in China. In the near future, data and information resources will become an important strategic resource and will be valued by people. It not only promoted the economic development of various regions and improved the industrial structure route but also promoted social and economic reforms nationwide.

The arrival of the era of big data has revolutionized the industrial structure of society. More than in the past, society values and relies on the holistic use of information. The essence of big data is the aggregation of complex data. Its basic mode of operation is the collection and processing of information [8]. Of course, the way big data technology collects and processes information is different from general data processing technology [9]. The data collected through big data technology can be sorted into valuable information in the hands of professionals, and the analysis and sorting of the collected data can also greatly improve the decision-making ability of managers [10]. The role of big data in organizational management, decision-making, and operation determines its practical value, and this value will continue to deepen and innovate. Its impact on organizational management has also become a symbol of the era of big data [11–14].

## 2. The Impact of the Urban Built Environment on the Physical and Mental Health of the Elderly

### 2.1. The Urban Built Environment Can Meet the Basic Living Needs of the Elderly

With the increase of age, the physical function of the elderly slowly declines, the ability to perceive things also begins to decline slowly, and the activity ability gradually weakens [15]. In addition, the elderly are more likely to suffer from various acute and chronic underlying diseases, and the devastation of diseases continues to accelerate the aging of the elderly, resulting in the deterioration of the physical and mental health of the elderly [16–20]. Therefore, the elderly are more likely to have fear and have higher expectations for living conditions and various social services [21]. They hope that they can live in a safer, more convenient, more comfortable, and healthier environment and receive more satisfactory daily care, medical and health protection, and spiritual and cultural comfort, as a guarantee for enjoying their old age and promoting the health of the elderly.

### 2.2. The Urban Built Environment Can Meet the Social Interaction Needs of the Elderly

The sense of loss, loneliness, change of social roles, and lack of social interaction after stopping work after retirement are problems that every elderly person needs to face and adapt to. As the mobility of the elderly gradually weakens, the social scope of the elderly gradually narrows [22–24]. The older the elderly, the smaller the range of activities and the fewer people they come into contact with. The study found that most of the current interpersonal communication patterns of the elderly are centered on relatives, and the elderly in their immediate neighbors are the main communication groups [25–27]. Relatives have begun to become the full support of the elderly, which brings about the extreme need of the elderly to express their emotions, they are eager to get more attention and love, and they are afraid of loneliness [28]. Through the built environment of the elderly city, improving the living environment and travel communication conditions of the elderly can help the elderly to improve their activity ability, expand the social circle for the elderly, help the elderly to exercise physically, make friends with the same age elderly people with similar interests, and enjoy each other. Appease, care, and help each other to promote the mental health of the elderly [29, 30].

### 2.3. The Urban Built Environment Can Meet the Social Participation Needs of the Elderly

With the end of career, old age begins to be freed from the burden of heavy social production and family economic creation. However, social and economic development has not completely separated the elderly, and the elderly themselves are not far from being completely separated from the social environment [31, 32]. They still care about social development and are willing to make progress together with society, develop their personal values, and realize self-dignity [33]. This demand is called social participation demand. The needs of social participation also have certain requirements on the built environment of elderly cities. In real life, the social participation of the elderly is mainly through participation in the community where they live, participating in some public welfare activities and entertainment activities organized by the community [34, 35]. The construction of the living environment for the elderly directly affects the social participation of the elderly. An urban built environment with complete supporting facilities and convenient public infrastructure can provide the necessary conditions for the social participation of the elderly and meet the needs of the elderly for social participation, thereby promoting the realization of the elderly [36–39].

## 3. Questionnaire Survey and Big Data Analysis

In order to analyze the factors affecting the physical and mental health of the elderly under the background of big data, this paper designs a questionnaire to investigate 2,000 elderly people living in urban areas [40]. The elderly conducted a questionnaire survey and distributed 1,000 random questionnaires to other relevant persons in the society for investigation. By extracting the key factors of the theory of personal health influencing factors in the urban construction environment theory, this paper defines the survey content of the questionnaire. The contents of these questionnaires are mainly about the relationship between the physical and mental health index of the elderly and the changes in the consensus degree of the urban built environment, urban built environment planning, urban built environment policy support, and multiparty efforts in the urban built environment. The main forms after the questionnaires are collected as follows.

From Table 1, it can be found that according to the range of the consensus degree [0, 1], the consensus degree of the elderly on the urban construction environment is not high, and they have doubts about various constructions.

From Table 2, it can be found that according to the range of planning participation [0, 1], the participation of the elderly in the urban construction environment is not high, and there is a situation of passive participation.

From Table 3, it can be found that according to the range of the approval degree [0, 1], the elderly have a low degree of approval for the urban construction environment, and there is a situation of environmental resistance.

From Table 4, it can be found that according to the range of the degree of cooperation between the elderly and the staff [0, 1], the relationship between the elderly and the coworkers of the urban construction environment is relatively common, and the probability of social people other than urban construction staff participating in this undertaking is low.

The following completes the data analysis of the reliability and validity of the questionnaire. First, reliability is tested, and overall reliability is measured by using Cronbach's coefficient. After scientific theoretical analysis, when Cronbach's coefficient is greater than 0.7 and the correlation of the statistical contents of each table is greater than one-third reliability. The reliability of this questionnaire is shown in Table 5.

The obtained tabular data are analyzed through Python and big data combined analysis formulas, and the combined analysis formulas involved are as follows:

(1) $$\begin{array}{c} \sigma =\sqrt{\frac{\sum_{i=1}^{n}{\left(x_{i}-\bar{x}\right)}^{2}f_{i}}{\sum_{i=1}^{n}f_{i}}},\\ \sigma^{2}=\frac{\sum_{i=1}^{n}{\left(x_{i}-\bar{x}\right)}^{2}f_{i}}{\sum_{i=1}^{n}f_{i}},\\ G=\sum \sqrt[{f}]{X_{1}^{f_{1}}\times X_{2}^{f_{2}}\times \ldots \times X_{n}^{f_{n}}}=\sum_{i=1}^{n}\sqrt[{f}]{\prod_{i=1}^{N}X_{i}^{f_{i}}},\\ \lambda =\sqrt{\frac{\sum_{i=1}^{d}{\left(\theta_{i}-\theta \right)}^{2}{\text{QYT}}_{i}}{YU}}. \end{array}$$

## 4. Problems Existing in the Built Environment of Elderly Cities

### 4.1. The Reconstruction Content Is Scattered and Lacks Comprehensive and Systematic Consideration

At present, there are still problems in the construction of the livable community environment for the elderly which mainly include the following points.

In the actual construction process, due to problems, such as weak foundation, limited funds, and complicated situations, the built environment or renovation of elderly cities is often simplified to adding ramps, handrails, and other fixed projects. These facilities are often not considered systematically, resulting in debris characterization. The urban built environment is an organic whole, including buildings, roads, landscapes, signs, lighting, and other aspects. At present, there are still some difficult problems in the living environment of the elderly, and the improvement of the environment needs to be strengthened urgently.

### 4.2. One-Sided Understanding of Needs, Ignoring the Needs of Other Groups

At present, many community builders have a one-sided understanding of the “livable elderly,” which is mainly manifested as follows: firstly, they separate the elderly from other groups and treat them independently, ignoring the complexity of the elderly's activities and the connection with other groups; secondly, it overemphasizes the needs of some seniors and ignores the needs of other groups in the community. These one-sided understandings make it difficult for the space environment built on it to match the real needs of the elderly.

### 4.3. The Design Is Rigid and Separated from the Actual Needs of the Elderly

Due to the lack of understanding of the concept of “living for the elderly” by some designers, there are many rigid cognitive misunderstandings in the actual construction process: first, simply equating “adapting to aging” with “accessibility”; second, ignoring the physical conditions of the elderly at different ages, not understanding the emotional needs of the elderly, and using some special facilities to connect the elderly with familiar social life status isolated.

## 5. Countermeasures and Suggestions for the Urban Built Environment for the Elderly

### 5.1. The Urban Built Environment for the Elderly Needs to Build Consensus

**Figure 1:**
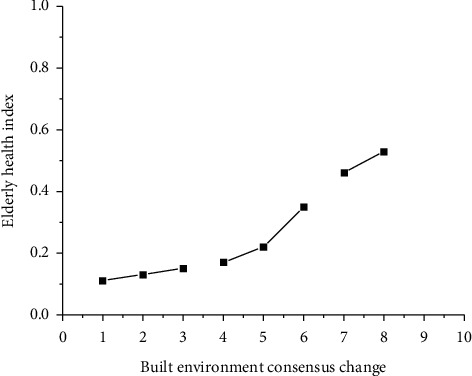
The relationship between the physical and mental health index of the elderly and the change of urban built environment consensus.

According to the analysis of the questionnaire and the big data formula, this paper obtained the results in Figure 1, and the concept determines the idea, and the idea determines the way out. Therefore, to do a good job in the built environment of elderly cities should be the concept first. We should focus on solving the deficiencies of public infrastructure and the urban and rural living environment in the construction of the built environment of elderly cities and solve the planning problems, supporting problems, and demand problems in urban construction. These current problems are not because the problems themselves are difficult to solve, but because of the lack of systematic concepts and lack of scientific foresight during the construction period. Therefore, the first problem to be solved now is the concept of the built environment of the elderly city. It is necessary to strengthen, popularize, and promote the concept of the built environment of the elderly city.

### 5.2. The Urban Built Environment for the Elderly Should Pay Attention to Construction Planning and Construction Standards

**Figure 2:**
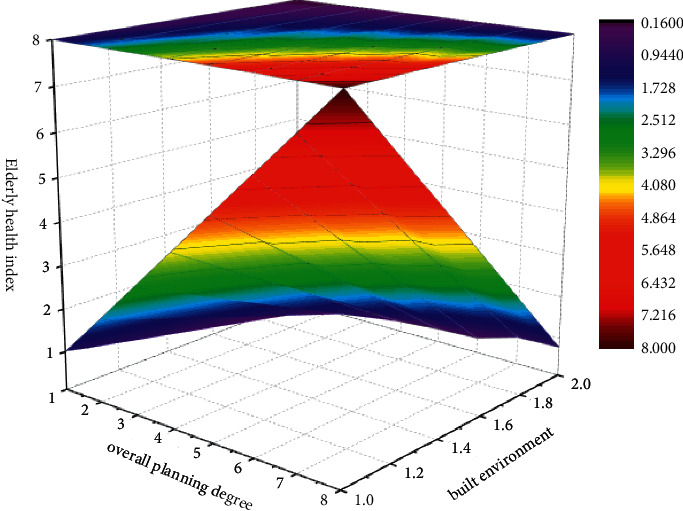
The relationship between the physical and mental health index of the elderly and urban built environment planning.

According to the analysis of the questionnaire and the big data formula, this paper obtained the results in Figure 2, and the built environment of the elderly city is a very meaningful work. The planning and the standard scale of the built environment of the elderly city need to be highly valued by relevant departments. Therefore, the author suggests that when government departments formulate urban and rural development and construction plans, they should integrate urban and rural public realm environment construction with the built environment of the elderly city. From the aspects of public infrastructure renovation, public safety and hygiene, life convenience, medical and health protection, spiritual and cultural needs, etc., we should scientifically plan, have a forward-looking layout, build supporting facilities suitable for the elderly, and explore the formation of a city suitable for the elderly to live in and have a convenient life.Requires compliance with environmental standards and regulations. At the same time, it is suggested that relevant departments should increase their efforts to support the construction of livable communities for the elderly, elderly care service institutions, and so on and other project construction efforts, construction projects to improve the quality of life of the elderly in the area, lasso centralized pension mode, and enrich the life and entertainment of the elderly.

### 5.3. The Construction of the Urban Built Environment for the Elderly Should be Combined with the Construction of Urban Livability, and Take This as an Opportunity for Further Development

**Figure 3:**
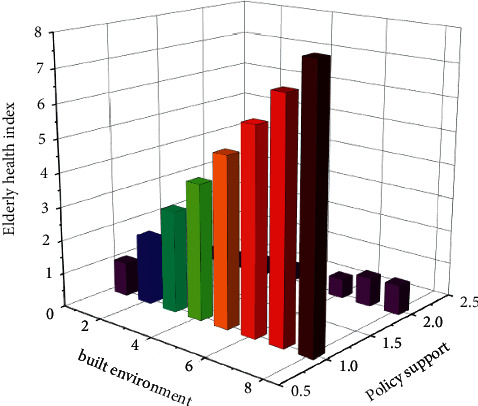
The relationship between the physical and mental health index of the elderly and the policy support of the urban built environment.

According to the analysis of questionnaires and big data formulas, this paper obtains the results in Figure 3. At present, all localities are vigorously carrying out urban livable construction work. The built environment of elderly cities should take this opportunity to comprehensively utilize the favorable policies related to urban livable construction to promote elderly cities. Built environment. According to the actual situation in various places, we adhere to the problem orientation, we keep an eye on the opportunities for the renovation of old communities and the improvement of the urban and rural environment, we devote ourselves to solving the most urgent practical difficulties of the elderly, and we vigorously carry out the work on the built environment of elderly cities. Through social transformation, strengthen the barrier-free transformation of the entrances and exits of the community, up and down ramps, elevators, stairs and other areas. Through various funding channels such as national special funds, collective investment by owners, community maintenance, and reconstruction funds, it is committed to the reconstruction of the public areas of the elderly living environment and helps the elderly to create an urban built environment with barrier-free homes and less travel difficulties.

### 5.4. The Construction of the Urban Built Environment for the Elderly Requires the Joint Efforts of People from All Walks of Life

**Figure 4:**
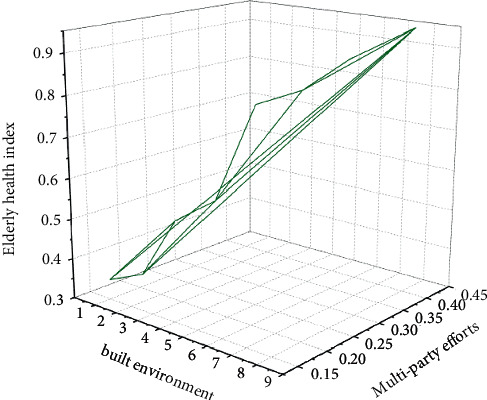
The relationship between the physical and mental health index of the elderly and the multiparty efforts in the urban built environment.

According to the analysis of questionnaires and big data formulas, this paper obtains the results in Figure 4. The built environment of elderly cities involves a wide range of departments and spans a wide range of professional fields. To do well in the cause of the built environment in elderly cities requires government agencies, market industries, and social organizations, family members, and members of wider society to work together, multiparticipation, to form a joint force. In addition, the cause of the built environment of elderly cities needs to be continuously explored and improved, and it needs to be forward-looking. Therefore, relevant departments and university researchers need to strengthen the research on the built environment of elderly cities, so as to provide theoretical and directional guidance for the development of the industry. Form the policy basis, industry standard scale and normative requirements of the industry. At the same time, all departments need to strengthen theoretical research and practical exploration according to the common problems and problems that need to be solved in the work of the built environment of elderly cities and constantly innovate and improve the related systems and mechanisms of the built environment of elderly cities.

## 6. Conclusion

The development of big data today is no longer a new thing. With the progress of society and the development of the times, the emergence of emerging technologies such as big data is an inevitable result. The concepts of “information explosion” and “mass data” were created in the information industry long before the concept of big data. Big data is not only large and complicated in operation but also means creating value for the society, mining potentially useful data from various fields for analysis and obtaining more value, which can further improve economic development and people's living standards.

The built environment and individual socioeconomic attributes are important factors affecting the health of the elderly in urban and rural areas. The increase in green exposure and the increase in the number of leisure facilities in the built environment not only reduces air pollution and noise pollution to a certain extent but also provides opportunities and venues for physical activity and social interaction for the elderly, thereby promoting the physical and mental health of the elderly in urban and rural areas. The improvement of road connectivity in the built environment promotes the proximity of the destination, optimizes the travel choices of the urban and rural elderly, provides convenience for their travel, enhances the desire of the urban and rural elderly to travel, and then conducts more healthy behaviors to promote their health. In addition, the increase in the number of intersections can slow down the speed of vehicles to a certain extent and provide safe walking conditions for the urban and rural elderly to travel. Among the socioeconomic attributes, the improvement of family income and culture, the growth of age, and the harmony of family atmosphere help to increase the investment in health of the elderly in urban and rural areas, so that the healthy behavior of the elderly can be more effectively guaranteed, thereby improving the health status.

There are differences in the influencing factors of the health status of the urban and rural elderly. First, the built environment has opposite effects on the physical activity of the urban and rural elderly, which in turn leads to differences in the health status of the urban and rural elderly. Second, the significance of socioeconomic attributes on the health of the urban and rural elderly is different. There are significant direct and indirect effects on the health of the urban elderly, and the direct effect on the health of the rural elderly is significant, but the indirect effect is not significant. Finally, the effect of physical activity on the health of the urban elderly is greater than that of the rural elderly, and the increase in physical activity can greatly improve the health of the elderly.

## Figures and Tables

**Table 1:** Survey on the consensus degree of the elderly and the urban construction environment.

	Park approval	Recognition of entertainment venues	Recreational facility approval
Urban elderly	0.43	0.35	0.42
Village elderly	0.32	0.23	0.36

**Table 2:** Survey on the elderly and urban construction environment planning.

	Do not understand planning	Clear planning	Participate in planning
Urban elderly	0.77	0.13	0.04
Village elderly	0.23	0.09	0.03

**Table 3:** Survey on the elderly and urban construction environment policy.

	Disagree policy	Agree	Strongly agree
Urban elderly	0.68	0.29	0.35
Village elderly	0.43	0.22	0.32

**Table 4:** Survey on the participation of other members of society in urban construction.

	Poor	Average	Very good
Urban elderly	0.32	0.33	0.25
Village elderly	0.26	0.21	0.11

**Table 5:** The reliability of the questionnaire.

Coefficient	Frequency		
	First questionnaire	Second questionnaire	Third questionnaire
Cronbach's coefficient	0.92	0.92	0.96
Relativity	0.65	0.80	0.85

## Data Availability

The datasets used and/or analyzed during the current study are available from the corresponding author on reasonable request.

## References

[1] Lu F., Tan S. (2015). Research on the influence of built environment on physical activity: progress and thinking. *International Urban Planning*.

[2] Lin X., Yang J. (2015). A review of the research on the relationship between built environment and public health in North American metropolitan areas and its enlightenment. *Planner*.

[3] Yang X., Sakar C., Webster C. (2017). The Relationship between Built Environment and Health Comes from an Exploratory Study of the High Density Healthy Cities. *Research Center of the University of Hong Kong Times Architecture*.

[4] Fei C., Zhou S., Zhang L. (2019). Influence of the built environment on residents’ leisure and physical activity from the perspective of life cycle. *World Geographic Research*.

[5] Yang C., Feng Z., Jiang Y. (2019). A research framework on the relationship between urban built environment and residents’ health based on an activity perspective. *Geographical Sciences*.

[6] Ma M., Zhou J., Cai Z. (2019). A review and enlightenment of health-oriented built environment and physical activity research. *Western Journal of Human Settlements*.

[7] Zhang Y., Deng W., Zhao L., Li M. (2020). How does the urban built environment affect the physical health of residents? ——intermediary mechanism and empirical Test. *Geographical Research*.

[8] Sun B., Yan H., Zhang T. (2016). The impact of community built environment on health: an empirical study based on individual overweight residents. *Acta Geographica Sinica*.

[9] Wang W., Feng Z., Jiang Y., Cai X. (2020). Study on the influence of built environment and individual characteristics on overweight and obesity in large city residents: taking Nanjing as an example. *Modern Urban Research*.

[10] Chun C., Hailili T., Chen Y. (2018). Research on the influencing factors of the built environment and planning responses to obesity in female elderly. *Human Geography*.

[11] Chun C., Chen X., Luo Z. (2020). Research on the impact of community built environment on respiratory health. *Planner*.

[12] Wang L., Jiang X., Sun W., Zhao X., Tang J. (2018). The impact of urban built environment on respiratory health and planning strategies: a city in Shanghai as an example. *Urban Planning*.

[13] Zhang Y., Qin B., Tang J. (2018). The impact of urban built environment on residents’ physical health based on propensity value matching method. *Acta Geographica Sinica*.

[14] Wang H., Cui D. (2020). Research on the built environment of Jinan residential community based on the characteristics of health needs. *Urban Development Research*.

[15] Zhang M., Tang Y., Liu Z., Yuan L. (2020). The great wall national cultural park: reshaping the relationship between the built environment and public health. *Beijing Planning and Construction*.

[16] Wang K. (2018). Research progress and enlightenment on the impact of built-up environment characteristics of urban parks on physical activity of users under the guidance of health. *Sport Science*.

[17] Sun B., Yin C. (2018). The impact of the built environment on the health of residents: evidence from demolition and resettlement housing residents. *Urban and Regional Planning Research*.

[18] Yu Y., Wang Q. (2018). Health-oriented construction of a livable environment for the elderly: international research progress and its enlightenment. *Urbanism and Architecture*.

[19] Yuan Y., Lin J., Xie L. (2018). Research progress on neighborhood impact of foreign residents’ health in the past 15 years: visual analysis based on CiteSpace software. *Tropical Geography*.

[20] Deeg D. J. H., Thomese G. C. F. (2005). Discrepancies between personal income and neighbourhood status:effects on physical and mental health. *European Journal of Ageing*.

[21] Phillipson C., Settersten R. A., Angel J. L. (2011). Developing age-friendly communities: new approaches to growing old in urban environments. *Handbook of Sociology of Aging*.

[22] Kamphuis C. B. M., Van Lenthe F. J., Giskes K., Huisman M., Brug J., Mackenbach J. P. (2009). Socioeconomic differences in lack of recreational walking among older adults:the role of neighbourhood and individual factors. *International Journal of Behavioral Nutrition and Physical Activity*.

[23] Broome K., Nalder E., Worrall L., Boldy D. (2010). Age-friendly buses? a comparison of reported barriers and facilitators to bus use for younger and older adults. *Australasian Journal on Ageing*.

[24] Siu V. W., Lambert W. E., Fu R., Hillier Ta, Bosworth M., Michael Yl (2012). Built environment and its influences on walking among older women: use of standardized geographic units to define urban forms. *Journal of Environmental and Public Health*.

[25] Thanakwang K., Isaramalai S. A. (2013). Productive engagement in older adults:a concept analysis. *Nursing and Health Sciences*.

[26] Scharlach A. E., Lehning A. J. (2013). Ageing-friendly communities and social inclusion in the United States of America. *Ageing and Society*.

[27] Buffel T., Phillipson C., Scharf T. (2012). Ageing in urban environments.Developing age-friendly cities. *Critical Social Policy*.

[28] Yur`Yev A., Leppik L., Tooding L. M. (2010). Social inclusion affects elderly suicide mortality. *International Psychogeriatrics*.

[29] Chen J., Wang Q., Huang J., Chen X. (2021). Motorcycle ban and traffic safety: evidence from a quasi-experiment at zhejiang, China. *Journal of Advanced Transportation*.

[30] Zheng W., Liu X., Yin L. (2021). Sentence representation method based on multi-layer semantic network. *Applied Sciences*.

[31] Yao L., Li X., Zheng R., Zhang Y. (2022). The impact of air pollution perception on urban settlement intentions of young talent in China. *International Journal of Environmental Research and Public Health*.

[32] Tang J. (2022). Rural residential elderly care community: a new scheme for the elderly in a suitable place. *Party and Government Research*.

[33] Lujunwei Y. M, Zhao C., Xu H. (2022). Exploration on local elderly care model in suxichang community. *Cooperative economy and science and technology*.

[34] Du S. H., Gong J., Gao H., Ye R., Guo S. Q. (2022). Analysis on the integration of care resources for urban home-based disabled elderly. *Health soft science*.

[35] Mei W. B. (2022). Research on the construction of evaluation index system for community aging environment construction. *Chinese Art*.

[36] Peng J., Zhang Y. F., Wang H., Ye G. M., Dong Y., Yin Q. (2022). Analysis on the health status and chronic disease comorbidity pattern of the long-lived elderly in Zhongxiang, Hubei. *Practical Preventive Medicine*.

[37] Hu D. B., Wang Y., Yang M., Li C. (2022). Study on extraction and antioxidant activity of polysaccharide from cephalosporium yunnanensis. *Chinese condiments*.

[38] Chen B. Y., Huang S., Liu Y. Q., Fan D. D., Li Y. (2022). Research on the art therapy of empty nest elderly care in community. *Heilongjiang human resources and social Security*.

[39] Gao N. (2022). Research on the old-age security of the accompanying elderly. *Heilongjiang human resources and social security*.

[40] Kang X., Zeng Y. J. (2022). Entrepreneurial bricolage based on big data and artificial intelligence decision-making. *Wireless Communications and Mobile Computing*.

